# An Omnidirectional Dual-Functional Metasurface with Ultrathin Thickness

**DOI:** 10.3390/ma15238378

**Published:** 2022-11-24

**Authors:** Ying Xiong, Xiaoyi Liu, Kai Wang, Xiaokun Wang, Xiaoyi Wang, Jinsong Gao, Haigui Yang

**Affiliations:** 1 Key Laboratory of Optical System Advanced Manufacturing Technology, Changchun Institute of Optics, Fine Mechanics and Physics, Chinese Academy of Sciences, Changchun 130033, China; 2 University of Chinese Academy of Sciences, Beijing 100039, China; 3 School of Optoelectronic Science and Engineering & Collaborative Innovation Center of Suzhou Nano Science and Technology, Soochow University, Suzhou 215006, China; 4 Key Lab of Advanced Optical Manufacturing Technologies of Jiangsu Province & Key Lab of Modern Optical Technologies of Education Ministry of China, Soochow University, Suzhou 215006, China

**Keywords:** metasurface, broadband high absorption, narrowband high emission, thermal light source, infrared imaging and detection

## Abstract

Although metasurfaces have received enormous attention and are widely applied in various fields, the realization of multiple functions using a single metasurface is still rarely reported to date. In this work, we propose a novel dual-functional metasurface that can be applied as a mid-infrared narrowband thermal light source in optical gas sensing and a long-wave infrared broadband absorber in photodetection. By actively tailoring the structure and constituent materials of the metasurface, the device yields an absorptivity of over 90% from 8 µm to 14 µm, while it exhibits an emissivity of 97.4% at the center wavelength of 3.56 μm with a full width at half-maximum of 0.41 µm. Notably, the metasurface is insensitive to the incident angle under both TM- and TE-polarized light. The proposed dual-functional metasurface possesses many advantages, including a simple structure, thin thickness, angle and polarization insensitivity, and compatibility with optical devices, which are expected to simplify the existing imaging systems and improve the performance of photodetection equipment.

## 1. Introduction

Metasurfaces are artificially constructed periodic sub-wavelength microstructures that can achieve a desired dielectric constant and permeability by actively selecting their material, shape, and arrangement so as to effectively manipulate the phase, amplitude, and polarization of electromagnetic waves [1,2,3,4]. Metasurfaces can precisely tune light–matter interactions at subwavelength scales, effectively excite surface plasmons (SPs), and achieve a variety of novel physical properties such as zero refractive index [5], negative reflection [6], super-resolution [7], and metalens [8,9,10]. More recently, many researchers have enabled metamaterials to achieve perfect emission/absorption by properly designing the electrical and magnetic responses, which break the limits of conventional devices and play important roles in the fields of thermal emitters [11], thermal imaging [12], energy harvesting [13,14], and gas detection [15], etc.

However, existing metasurfaces usually exhibit a single function [16,17,18]. For instance, detecting and monitoring hazardous gases has an important role in health, safety, and environmental protection [19,20,21]. Each gas has its own characteristic absorption spectrum in the infrared (IR) band. Highly selective, sensitive, compact, and low-cost sensing devices can be accordingly developed by designing narrowband high-emission metasurfaces corresponding to the characteristic spectra of the target gases; on the other hand, long-wave infrared (LWIR) detection is limited by the thickness of conventional absorbers. Distinct from omnidirectional absorbers with 1D multilayers or 2D or dispersed structures in a wide wavelength range [22,23,24], metasurfaces can effectively break the limitations of traditional materials and significantly improve the performance of LWIR detectors [25,26,27]. In general, the working waveband and function of a metasurface are already determined during the design process, while integrating multiple functions in different wavebands usually needs a metasurface group, undoubtedly increasing the complexity and difficulty of the design. Although challenging, it would be highly desirable if a single metasurface could be developed to achieve multiple functions, bringing great advantages in terms of both expanding applications and simplifying manufacturing steps.

In this work, we aim to combine two functions of gas sensing and detection together. We propose a novel dual-functional metasurface featuring mid-infrared (MIR) narrowband thermal emission and long-wave infrared broadband absorption simultaneously. Compared with other metasurfaces, higher emission/absorption can be achieved by optimizing the resonator geometry in combination with high-loss materials. The design process and the emission/absorption properties of the metasurface are specifically demonstrated and simulated. Then, to analyze the principle of its emission/absorption generation, the electromagnetic field distribution in the microstructure is calculated and discussed. An equivalent circuit model is proposed to explain the corresponding principle. Subsequently, to validate the feasibility of its two applications in optical gas sensing and LWIR detection, the emission performance of the proposed metasurface is tested under different incident angles and polarizations. Finally, a brief outlook on its development potential is presented.

## 2. Structure Design and Simulation Details

To achieve high emission/absorption, the metasurface needs to suppress reflection and transmission in the operating wavelength range, and to this end, we conducted a series of designs in terms of material selection, structural design, and numerical simulation.

Metasurfaces are usually composed of three parts: a resonator, spacer, and bottom layer. We used chromium (Cr) as the material for the resonator and bottom layer. The intrinsic broadband plasmonic resonances of Cr can broaden the bandwidth of absorption, and the inherent loss of the metal can further improve the intensity of emission and absorption [28]. The thicknesses of the bottom layer and resonator were both 100 nm, which can suppress the transmission and excited localized surface plasmons (LSPs) to enhance the absorption of the metasurface, respectively. On the other hand, high-dispersive dielectrics, such as silicon dioxide (SiO_2_) and silicon nitride (Si_3_N_4_), have intrinsic vibrational modes (optical phonons) in the infrared range [29]. These optical phonons can be coupled with excited plasmonic effects, thereby modifying the optical response of the metasurface. Accordingly, we selected SiO_2_ and Si_3_N_4_ as the spacer of the metasurface.

**Figure 1:**
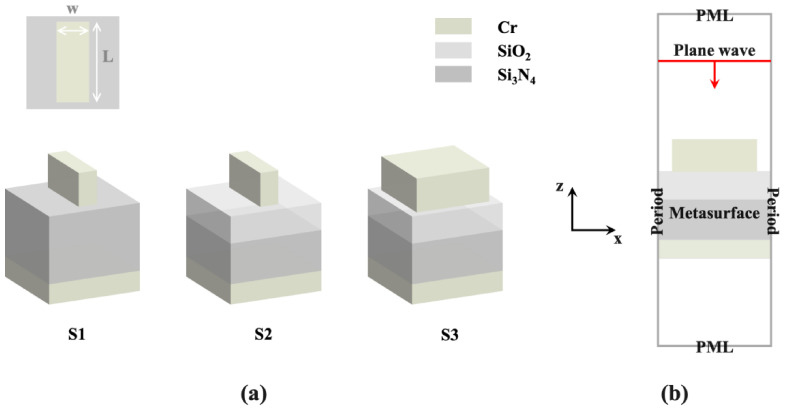
(**a**) Design process of metasurfaces involving three structures of S1, S2, and S3. (**b**) Schematic of boundary condition settings in simulation.

In terms of structural design, we first developed a Cr–Si_3_N_4_–Cr metasurface S1, which was comprised of metal wire resonators, a metal plane bottom, and a dielectric layer in the middle. On this basis, we selected SiO_2_ to overlay the spacer layer to build a new microstructure S2. To confine its operating wavelengths in a certain range, we adjusted the parameters of the resonator by performing numerical simulations. By actively choosing the resonator structure, optical resonances within the metasurface were effectively enhanced. Finally, a square resonator geometry was designed. To shift the emission peak of the metasurface towards 3.58 μm to match the characteristic spectrum of CH_4_ gas, we adjusted the composition of the dielectric layer. After sufficient simulations for structure parameter optimization, we chose a stacked dielectric layer consisting of SiO_2_ and Si_3_N_4_ with thicknesses of 80 nm and 720 nm, respectively. The new Cr–SiO_2_–Si_3_N_4_–Cr metasurface was named S3. The structural diagrams of S1, S2, and S3 are shown in Figure 1a.

The metasurface was numerically simulated by the finite-difference time-domain (FDTD) algorithm. One unit cell of the proposed periodic microstructure was simulated. Each simulation typically took 2–3 h by using a common personal computer. In a series of simulations, the periodic condition was adopted as the boundary condition in both X- and Y-directions, while the perfectly matched layer condition was adopted in the Z-direction for the sake of extracting the reflectance spectra (see Figure 1b). The characteristic parameters for the Cr and SiO_2_ material such as the permittivity and refractive index, were obtained from the Palik database [30], and the Si_3_N_4_ parameters were obtained by Kischkat [31]. The absorptance (A) of the metasurface was calculated through the reflectance (R) and transmittance (T) obtained by simulation: A = 1 − R − T. The simulated emissivity was subsequently obtained according to Kirchhoff’s law of thermal radiation, namely, the emissivity of material equals its absorptivity at thermodynamic equilibrium. Additionally, the simulations were repeated to guarantee their accuracy.

## 3. Results and Discussion

### 3.1. Optical Prorperties of Designed Metasurfaces

**Figure 2:**
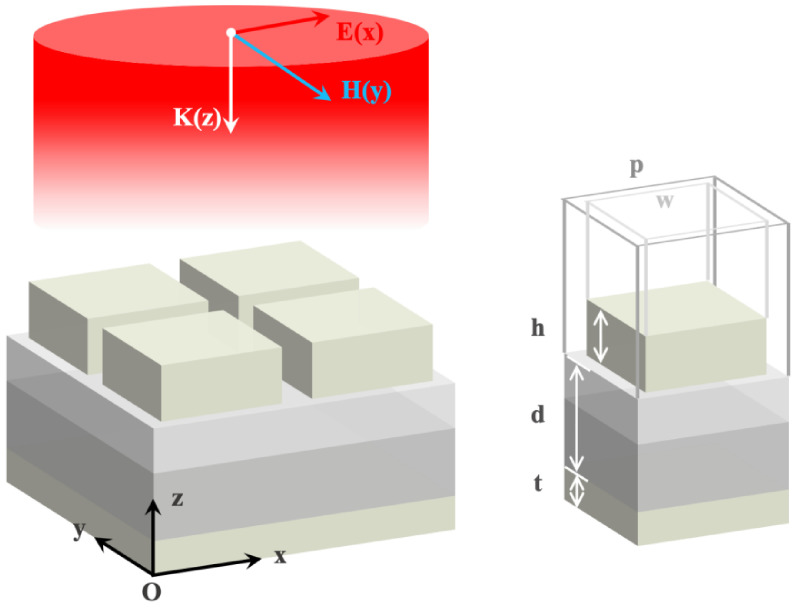
Diagram of designed metasurface S3 with *p* = 1000 nm, w = 800 nm, h = 100 nm, d = 800 nm, and t = 100 nm.

**Figure 3:**
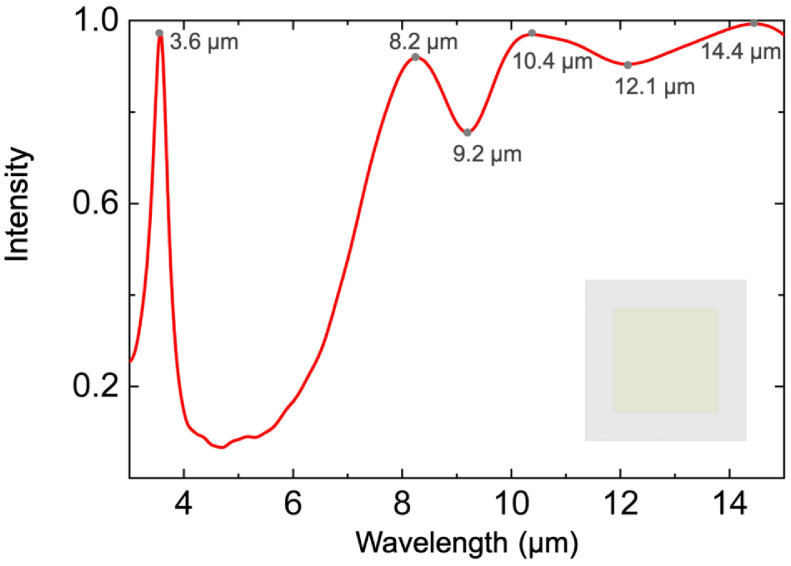
Simulated emission/absorption spectrum of the designed metasurface S3. The inset is the vertical view of the metasurface structure.

Table 1 exhibits the fundamental parameters and numerical simulation results of the three mentioned structures. L and w are the length and width of the resonator, respectively. After continuous optimization, the average emissivity of the metasurface in the entire LWIR is improved from 81.3% to 92.0%, and the maximum emissivity in MIR is up to 97.4%. To concretely investigate the features of structure S3, we illustrate its detailed structural parameters in Figure 2. The thicknesses of the resonator, dielectric layer, bottom layer, and period are denoted as h, d, t, and p, respectively. Figure 3 shows the calculated emissivity/absorptivity spectrum of S3. There is a high absorption of 92.0% in a broadband wavelength spread from 8 to 14 µm, covering the entire LWIR atmospheric window. The simulated absorption spectrum has three peaks at wavelengths of 8.2, 10.4, and 14.4 µm with absorptivities of 92.8%, 96.6%, and 99.3%, respectively. Moreover, a high emissivity of 97.4% is achieved at a wavelength around 3.6 µm with a full width at half-maximum (FWHM) of 0.41 µm in MIR. In brief, a narrowband high emission in MIR and a broadband high absorption in LWIR can be simultaneously achieved by the proposed structure S3.

### 3.2. Principle of Metasurface Emission/Absorption Generation

**Figure 4:**
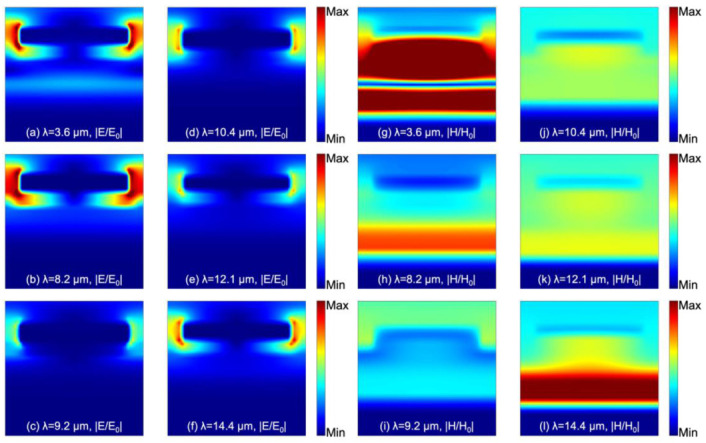
Electric field distribution of S3 at (**a**) λ = 3.6 µm, (**b**) λ = 8.2 µm, (**c**) λ = 9.2 µm, (**d**) λ = 10.4 µm, (**e**) λ = 12.1 µm, and (**f**) λ = 14.4 µm. The color bar indicates the relative electric field intensity (E/E_0_). Magnetic field distribution at (**g**) λ = 3.6 µm, (**h**) λ = 8.2 µm, (**i**) λ = 9.2 µm, (**j**) λ = 10.4 µm, (**k**) λ = 12.1 µm, and (**l**) λ = 14.4 µm. The color bar indicates the relative magnetic field intensity (H/H_0_).

**Figure 5:**
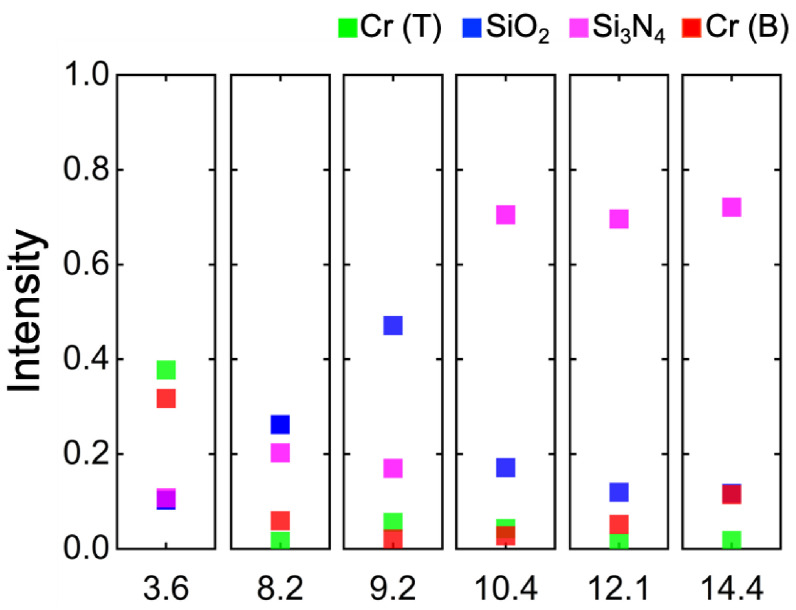
Intensity distribution of the metasurface S3 for each layer at 3.6 µm, 8.2 µm, 9.2 µm, 10.4 µm, 12.1 µm, and 14.4 µm.

To analyze the principle of the emission/absorption generation, we calculated and analyzed the electromagnetic field distribution of S3 at 3.6 µm, 8.2 µm, 9.2 µm, 10.4 µm, 12.1 µm, and 14.4 µm (see Figure 4). Meanwhile, we also calculated its intensity distribution for each layer at the above wavelengths using the following equation (see Figure 5) [32]:

(1) Q(ω)=12×ω×Im(ε)×E2(ω)

in which ω is the angular frequency, Im(ε) is the imaginary part of the dielectric permittivity, and E(ω) is the local electric field. Here, the electric field intensity used in the calculation is based on the relative values extracted from the simulation results in Figure 4a–l; thus, Q(ω) calculated by Equation (1) is the relative distribution rather than the actual one.

Through the distribution of the electric field presented in Figure 4a–f, we found that the current is mainly concentrated at the edges of the resonator. It can be suggested that the LSPs are excited at the edges of the resonator due to the electromagnetic wave incident on the metamaterial; however, it is clear that they do not have the same intensity since the response intensity is related to the material, shape, and size of the resonators.

To further explain the physical mechanism, we simulated the magnetic field distribution of the metasurface at each wavelength. The first mode is M1, as shown in Figure 4j–l. The PSP excited at the dielectric—metal interface enables the magnetic field to be mainly concentrated below the resonator. Then, the SPs will be coupled to the dielectric layer interacting with the optical phonons of the Si3N4 layer, and the coupling will be further enhanced in the cavity consisting of the resonator and the bottom layer. The optical property of the PSP can be expressed theoretically by the following equations [33]:

(2) $$k=k_{0}\sin\theta +i\times \frac{2\pi }{p}$$

(3) $$k_{PSP}=k_{0}\sqrt{\frac{\varepsilon_{m}\varepsilon_{d}}{\varepsilon_{m}+\varepsilon_{d}}}$$

Equation (2) is the Bragg coupling condition, in which $k_{0}=\frac{\omega }{C}=\frac{2\pi }{\lambda }$ is the free-space wave vector, θ is the angle of the incident electromagnetic wave, integer i is the diffraction order of grating, and p is the grating period. Equation (3) indicates the wave vector of the PSPs; $\varepsilon_{m}$ and $\varepsilon_{d}$ are the dielectric constants of the metal and insulator medium, respectively. Only when $k=k_{PSP}$ does the incident electromagnetic wave couple to PSPs.

The second mode is M2, as shown in Figure 4h,i. The magnetic field is mainly distributed in the dielectric layer, which is attributed to the strong coupling of the LSP generated by the resonator with the optical phonons in the dielectric. As shown in Figure 4, the SiO_2_ in the stacked dielectric layer contributed to the absorption the most.

**Figure 6:**
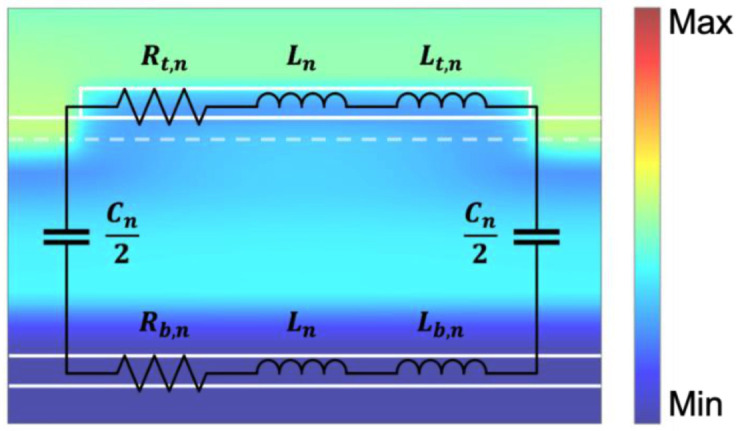
Description of the LSPR mode at the metasurface S3. Equivalent circuit model of the resonant mode at a magnetic field distribution with a normal incidence angle resonance wavelength of 9.2 µm. The color bar indicates the relative magnetic field intensity (H/H_0_).

The LSPR mode of the metasurface can be described by the equivalent circuit model. As shown in Figure 6a, the resonance wavelength $\lambda_{n}$ can be expressed as [34]:

(4) $$\lambda_{n}=2\pi cC_{n}\sqrt{2L_{n}+L_{t,n}+L_{b,n}}$$

The parameters of the equivalent circuit model indicated in Figure 6 can be obtained such that $L_{n}=\frac{0.5\mu_{0}dl}{nw}$ denotes the inductances of the resonator and bottom layer separated by the dielectric spacer; $C_{n}=\frac{2\beta_{n}\varepsilon_{0}\varepsilon_{d}lw}{nd}$ is the capacitance of the dielectric spacer between the two parallel plates; $R_{t,n}=\frac{l}{n\delta_{\mathrm{eff}}w\omega \varepsilon_{0}}\left(\frac{\varepsilon_{2}}{\varepsilon_{1}^{2}+\varepsilon_{2}^{2}}\right)$ and $L_{t,n}=\frac{-l}{n\delta_{\mathrm{eff}}w\omega^{2}\varepsilon_{0}}\left(\frac{\varepsilon_{1}}{\varepsilon_{1}^{2}+\varepsilon_{2}^{2}}\right)$ are the resistance and kinetic inductances of the resonator, respectively; and $R_{b,n}=\frac{1}{n\delta_{\mathrm{eff}}\omega \varepsilon_{0}}\left(\frac{\varepsilon_{2}}{\varepsilon_{1}^{2}+\varepsilon_{2}^{2}}\right)$ and $L_{b,n}=\frac{-1}{n\delta_{\mathrm{eff}}\omega^{2}\varepsilon_{0}}\left(\frac{\varepsilon_{1}}{\varepsilon_{1}^{2}+\varepsilon_{2}^{2}}\right)$ are the resistance and kinetic inductances of the bottom layer, respectively. For these parameters, the resonance modes of the metasurface are labeled with the subscript n; ω is the angular frequency of the incident wave; $\mu_{0}$ and $\varepsilon_{0}$ are the vacuum permeability and vacuum permittivity, respectively; $\varepsilon_{d}$ is the dielectric permittivity; $\varepsilon_{1}$ and $\varepsilon_{2}$ are the real and imaginary parts of the metal permittivity, respectively; $\beta_{n}$ considers the non-uniform charge distribution along the surfaces of the capacitor; $\delta_{\mathrm{eff}}$ is the effective penetration depth of the metal; and c denotes the speed of light in the vacuum. It can be said that, in the above two modes, the local plasmon resonance (LSPR) of the resonator, the cavity resonance between the resonator and the substrate, and the optical phonon resonance in the spacer are the main factors required for the proposed metasurface to achieve the near-perfect absorption characteristics in the broadband range.

The third mode is M3, as shown in Figure 4g. For the high emissivity at 3.6 µm (Figure 5), we found that the metal made the largest contribution to the emission, which is attributed to the intrinsic plasmon resonance and inherent loss of the metal, in agreement with our mentioned design concept. Due to the Fabry–Perot cavity between the resonator and bottom layer, the LSPR excited by the resonator is further enhanced, which significantly contributes to the high emissivity of the metasurface.

As previously mentioned, the principle of emission/absorption generation in the metasurface is consistent with our original design, and furthermore, different resonance modes will promote or inhibit each other. For example, in the LWIR region, the interactions between optical phonons and SPs in different dielectric layers (SiO_2_ and Si_3_N_4_) can be mutually suppressed or not acted upon owing to their different response bands, which ultimately broadens the absorption wavebands of the proposed metasurface.

### 3.3. Metasurface Performance under Different Incident Conditions

**Figure 7:**
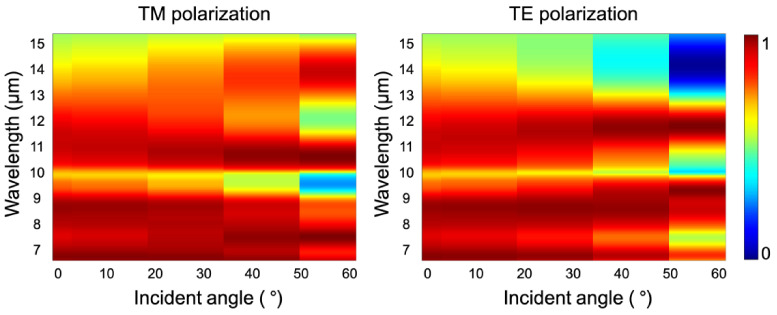
Absorption performance of the metasurface under different incident angles and polarization statuses. The color bar indicates the absorption intensity.

In consideration of the performance stability of the metasurface within a wide range of incident angles and polarization statuses, we characterized the dependence of its absorption performance on different incident conditions, as shown in Figure 7. The simulated results show that the broadened high absorptivity of the proposed metasurface in the LWIR region can be maintained when the incident angle varied from 0° to 40° under TE-polarized light and up to 50° under TM-polarized light. Thus, the performance of the designed metasurface remains efficient and stable under different incident conditions, i.e., exhibits “omnidirectional” features, which is beneficial for realizing dual applications in the areas of gas sensing and LWIR detection.

**Figure 8:**
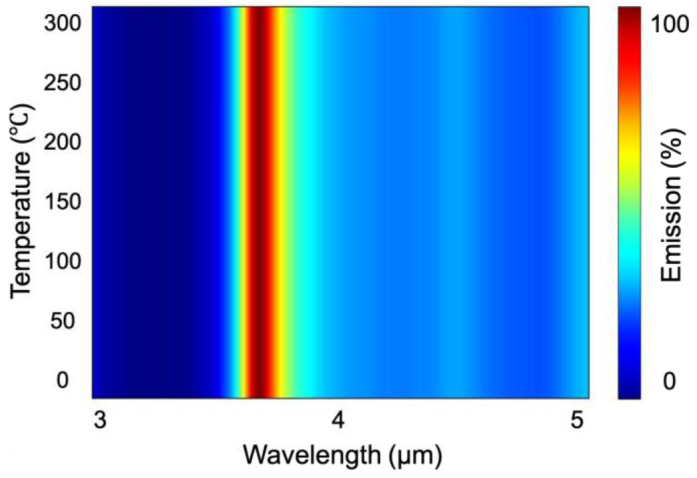
Thermal stability of the metasurface. The color bar indicates the emission intensity.

The absorption of the metasurface will inevitably lead to an increase in temperature. Therefore, we investigated the emission performance of the metasurface at different temperatures. As shown in Figure 8, the proposed metasurface presents excellent thermal stability, as indicated by its ability to monitor the target gas with high efficiency even at an extreme temperature (300 °C).

In order to underline the unique working wavebands of the proposed metasurface, we provide information about several dual-band metamaterials previously reported in the literature as a contrast, as shown in Table 2.

**Figure 9:**
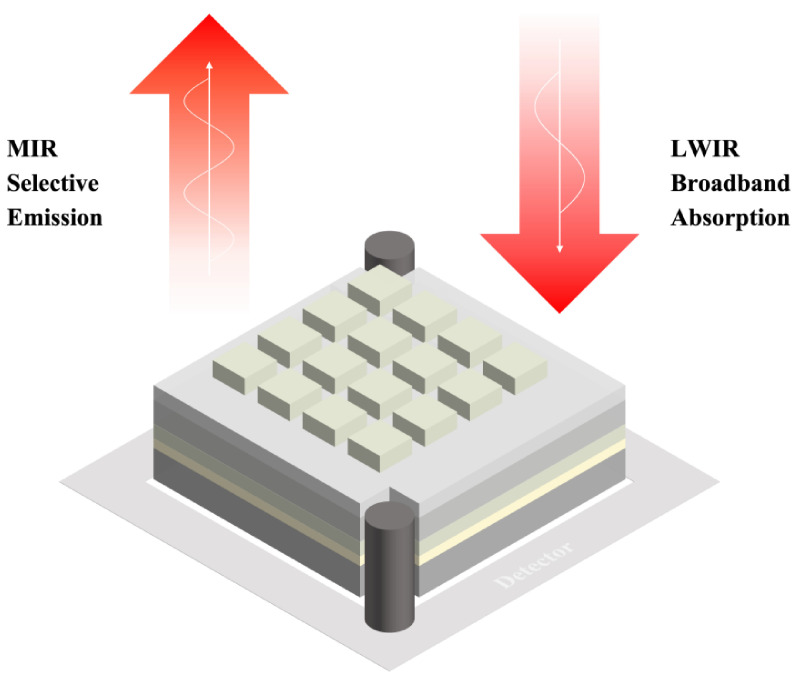
Demonstration of the designed dual-function metasurface.

Figure 9 gives a visual schematic of the dual functions of the proposed metasurface. In brief, the advantages of the proposed metasurface as compared with conventional metasurfaces are:

(i). Narrowband high-emission (FWHM as low as 0.41 µm), which can be utilized as a compact selective thermal light source for gas sensing;
(ii). High absorptivity in broadband (over 90% from 8 to 14 µm), which can be applied as a LWIR broadband absorber in photodetectors;
(iii). Great absorption/emission stability under large incident angles and different polarization statuses;
(iv). Ultra-thin thickness (T = 1 µm), which is ideal for directly integrating with detectors and is of great benefit in improving its photodetection performance.

With the development of microfabrication technologies, the fabrication of metamaterials is feasible. Specifically, the multilayer structure of the proposed metamaterials can be prepared by magnetron sputtering or electron beam evaporation, while the square resonator can be prepared by existing nano-processing methods such as electron beam lithography (EBL).

## 4. Conclusions

In summary, we have successfully demonstrated a novel wide-angle, dual-function metasurface with simple structure and thin thickness. The combination of LSPR, PSPR, and optical phonon resonance on the metasurface achieves a high absorptivity of over 90% in the broadband range. Meanwhile, the intrinsic plasmonic resonances and the inherent loss of the metal in the metasurface leads to a narrowband high-emissivity of 97.4% with an FWHM of 0.41 µm due to the Fabry–Perot resonance between the resonator and bottom layer. These characteristics permit the designed metasurface to simultaneously act as a compact selective thermal source in gas sensing and an LWIR broadband absorber in photodetectors. The development of a dual-functional metasurface with a simple configuration such as this is highly desirable and significant.

## Figures and Tables

**Table 1:** Fundamental structural parameters and numerical simulation results of S1, S2, and S3.

Metasurface	Material Selection	Geometrical Parameters	SiO_2_ Thickness	Si_3_N_4_ Thickness	Emission Peak Wavelength	Maximum Emissivity in MIR	Average Absorptivity in LWIR
S1	Cr Si_3_N_4_	L = 800 nm w = 200 nm	0 nm	1000 nm	4.06 μm	86.4%	81.3%
S2	Cr SiO_2_ Si_3_N_4_	L = 800 nm w = 200 nm	80 nm	720 nm	3.58 μm	86.6%	84.2%
S3	Cr SiO_2_ Si_3_N_4_	L = 800 nm w = 800 nm	80 nm	720 nm	3.58 μm	97.4%	92.0%

**Table 2:** Comparison of optical properties of representative infrared dual-band metasurfaces in recent years.

References	MIR			LWIR		
	Average Absorptivity	Central Wavelength	Absorption Bandwidth	Average Absorptivity	Central Wavelength	Absorption Bandwidth
[35]	85.0%	6.18 μm	narrowband	89.0%	8.32 μm	narrowband
[36]	>80.0%	6.15 μm	2.70 μm	>80.0%	10.10 μm	0.80 μm
[37]	>90.0%	3.70 μm	narrowband	>90.0%	11.20 μm	narrowband
[38]	80.0%	approximate 4.20 μm	0.38 μm	>90.0%	approximate 10.40 μm	2.48 μm
[39]	50.0%	4.00 μm	2.00 μm	<90.0%	10.50 μm	5.00 μm
our work	97.4%	3.58 μm	0.82 μm	92.0%	11.00 μm	6.00 μm

## Data Availability

The authors confirm that the data supporting the findings of this study are available within the article.
